# Metagenomic- and Cultivation-Based Exploration of Anaerobic Chloroform Biotransformation in Hypersaline Sediments as Natural Source of Chloromethanes

**DOI:** 10.3390/microorganisms8050665

**Published:** 2020-05-02

**Authors:** Peng Peng, Yue Lu, Tom N.P. Bosma, Ivonne Nijenhuis, Bart Nijsse, Sudarshan A. Shetty, Alexander Ruecker, Alexander Umanets, Javier Ramiro-Garcia, Andreas Kappler, Detmer Sipkema, Hauke Smidt, Siavash Atashgahi

**Affiliations:** 1 Laboratory of Microbiology, Wageningen University & Research, 6708 WE Wageningen, The Netherlands; peng677@gmail.com (P.P.); yuelu@hnu.edu.cn (Y.L.); tom1.bosma@wur.nl (T.N.P.B.); sudarshan.shetty@wur.nl (S.A.S.); aleksandr.umanetc@wur.nl (A.U.); javier.ramirogarcia@uni.lu (J.R.-G.); detmer.sipkema@wur.nl (D.S.); hauke.smidt@wur.nl (H.S.); 2 College of Environmental Science and Engineering, Hunan University, Changsha 410082, China; 3 Key Laboratory of Environmental Biology and Pollution Control, Hunan University, Ministry of Education, Changsha 410082, China; 4 Helmholtz Centre for Environmental Research-UFZ, Department of Isotope Biogeochemistry, 04318 Leipzig, Germany; ivonne.nijenhuis@ufz.de; 5 Laboratory of Systems and Synthetic Biology, Wageningen University & Research, 6708 WE Wageningen, The Netherlands; bart.nijsse@wur.nl; 6 Department of Biogeochemical Processes, Max Planck Institute for Biogeochemistry, 07745 Jena, Germany; aruecker@bgc-jena.mpg.de; 7 Geomicrobiology, Centre for Applied Geosciences, University of Tuebingen, 72076 Tuebingen, Germany; andreas.kappler@uni-tuebingen.de

**Keywords:** hypersaline lakes, chloroform, biotransformation, metagenome

## Abstract

Chloroform (CF) is an environmental contaminant that can be naturally formed in various environments ranging from forest soils to salt lakes. Here we investigated CF removal potential in sediments obtained from hypersaline lakes in Western Australia. Reductive dechlorination of CF to dichloromethane (DCM) was observed in enrichment cultures derived from sediments of Lake Strawbridge, which has been reported as a natural source of CF. No CF removal was observed in abiotic control cultures without artificial electron donors, indicating biotic CF dechlorination in the enrichment cultures. Increasing vitamin B_12_ concentration from 0.04 to 4 µM in enrichment cultures enhanced CF removal and reduced DCM formation. In cultures amended with 4 µM vitamin B_12_ and ^13^C labelled CF, formation of ^13^CO_2_ was detected. Known organohalide-respiring bacteria and reductive dehalogenase genes were neither detected using quantitative PCR nor metagenomic analysis of the enrichment cultures. Rather, members of the order *Clostridiales*, known to co-metabolically transform CF to DCM and CO_2_, were detected. Accordingly, metagenome-assembled genomes of *Clostridiales* encoded enzymatic repertoires for the Wood-Ljungdahl pathway and cobalamin biosynthesis, which are known to be involved in fortuitous and nonspecific CF transformation. This study indicates that hypersaline lake microbiomes may act as a filter to reduce CF emission to the atmosphere.

## 1. Introduction

Until the 1970s, halogenated organic compounds, organohalogens, were believed to originate exclusively from anthropogenic sources [1]. This long-held view was changed following the discovery of diverse organohalogens from natural environments. To date, over 5000 organohalogens with natural origins have been identified [2]. A remarkable example is chloroform (CF), a known environmental contaminant and a potential carcinogen that bioaccumulates in living organisms with harmful impacts [3]. CF is synthetically produced in chemical industries for various applications [4]. However, overall anthropogenic sources were estimated to contribute to less than 10% of the global CF production of 700–820 Gg/y [5]. Natural CF emissions have been reported from numerous terrestrial and aquatic environments such as forest soils [6,7,8,9], rice fields [10], groundwater [11], oceans [12], and hypersaline lakes [13,14]. The formation of CF is mediated by biotic and abiotic processes, including burning of vegetation, chemical production by reactive iron species, and enzymatic halogenation [15]. Similar to other low molecular weight volatile organohalogens (VOX, e.g., chlorofluorocarbons), CF release into the atmosphere can cause ozone depletion and impact climate change [16]. 

CF is persistent in the environment and is hardly dechlorinated/degraded under oxic conditions [17,18], whereas many microbes can transform CF in the absence of oxygen [19,20,21,22,23]. Anaerobic CF transformation has been reported for acetogens like *Acetobacterium woodii* [24] and *Clostridium* sp. [25], methanogenic *Methanosarcina* spp. [26,27,28], and fermentative *Pantoea* spp. [23]. These microbes transform CF to dichloromethane (DCM), carbon monoxide (CO), and/or carbon dioxide (CO_2_). CF transformation by acetogens and methanogens is a co-metabolic process, even though the responsible genes and enzymes are not yet clear. Previous studies suggested that enzymes involved in the Wood-Ljungdahl pathway (WLP) and methanogenesis may mediate co-metabolic CF transformation [24,29]. Additionally, transition-metal co-factors such as cob(I)/cob(II)alamins (reduced form of vitamin B_12_ (cob(III)alamins)) and F_430_ (nickel(I)-porphinoid), which are required by the key enzymes of acetogenesis and methanogenesis, e.g. methyltransferase and methyl-coenzyme M reductase, respectively, can act as reductants and nucleophilic reagents and catalyze fortuitous and nonspecific reductive dechlorination of chloromethanes [30,31,32].

Another group of anaerobes known as organohalide-respiring bacteria (OHRB) can use CF as a terminal electron acceptor and couple CF reductive dechlorination to energy conservation [33,34]. For instance, CF respiration to DCM has been reported for *Desulfitobacterium* sp. strain PR [35], *Desulfitobacterium hafniense* TCE1 [36], *Dehalobacter* sp. strain UNSWDHB [37,38], and a mixed culture containing *Dehalobacter* [21]. The enzymes responsible for reductive dehalogenation in OHRB are mainly corrinoid-dependent reductive dehalogenases (RDases). One CF RDase (CfrA) has been identified from *Dehalobacter*-containing microbial consortia [39]. CF can also be abiotically dechlorinated under anoxic conditions to DCM, via hydrogenolysis, or to CH_4_, via reductive elimination [40,41,42].

Previous studies have shown the presence of organohalogen-metabolizing microbes in environments where natural organohalogens have been shown or suspected to be present [43,44], indicating a potential interdependency between halogenation and dehalogenation [45]. Hypersaline lakes are natural sources of VOX, and (micro)organisms are major contributors of VOX emission in these environments [13,14,46]. Moreover, NaCl in hypersaline lakes might promote high rates of organic matter halogenation [47]. Such natural production may in turn promote development of biochemistries for VOX transformation. However, knowledge about the microbial metabolism of VOX in such extreme environments is lacking. This information is necessary to understand whether microbes in hypersaline lakes can act as a filter to reduce VOX release to the atmosphere. [13]. The aim of this study was therefore to investigate CF transformation potential and underlying microorganisms/pathways in sediments of two hypersaline lakes in Western Australia, i.e., Lake Strawbridge and Lake Whurr. To this end, we used a combination of anaerobic cultivation in microcosms, metabolite analyses, stable isotope labelling, molecular analyses, and genome-resolved metagenomics. Anoxic microcosms were prepared from the sediments of both lakes, but CF transformation was only noted in the microcosms of Lake Strawbridge, which was interestingly reported as a natural source of CM and CF [13]. Since CF (co-)metabolism under anoxic conditions is usually vitamin B_12_-dependant [33,48], we tested the impact of different vitamin B_12_ concentrations on CF transformation. Metagenomic (and molecular) analyses were done to identify the OHRB-harboring RDases genes/enzymes responsible for metabolic CF dechlorination, or the enzymatic repertoires needed for co-metabolic CF transformation, such as the WLP and cobalamin biosynthesis routes [20,48]. We were able to show the occurrence of CF transformation in hypersaline lakes, verified the lack of known OHRB and RDase genes, and identified the WLP and cobalamin synthesis pathways essential for co-metabolic CF transformation.

## 2. Materials and Methods

### 2.1. Sediment Sampling

Duplicate sediment cores of approximately 24 cm length and 4 cm internal diameter were collected from Lake Strawbridge (LS, 32.84°S, 119.40°E) and Lake Whurr (LW, 33.04°S, 119.01°E) in Western Australia (Supplementary Figure S1). Sediment cores were taken by pushing a polypropylene tube into the lake sediment. The top and the bottom of the tube were immediately closed with rubber stoppers after pulling the core from the sediment. The sediment cores were transported at 8 °C to the Laboratory of Microbiology, Wageningen University & Research, The Netherlands. 

### 2.2. Physical Chemical Analysis

Upon arrival at the laboratory, the sediment cores were cut into a top (0–12 cm) and a bottom (12–24 cm) layer in an anoxic chamber filled with an atmosphere of N_2_/H_2_ (96%/4%). Subsamples from each sediment layer were homogenized and subsequently used for physical chemical analysis and as inocula for microcosm preparation. The remaining sediments were kept at -80 °C for molecular and metagenomic analysis.

Water content was determined by the percentage of weight loss observed after drying the samples overnight at 105 °C in an oven, followed by cooling down to room temperature in a desiccator. pH was measured from air-dried sediments suspended in 0.01 M CaCl_2_ solution after two hours using a pH meter (ProLine B210, Oosterhout, The Netherlands). Sediment total organic carbon (TOC) was measured using the Kurmies method [49]. Low crystalline iron was extracted from 0.5 g wet sediment using 25 mL of 0.5 M anoxic HCl for one hour in the dark [50], and concentrations of dissolved Fe(II) and Fe(III) were quantified uisng spectrophotometric determination with ferrozine [51]. Major anions including Cl^-^, SO_4_^2-^, NO_3_^-^, and ClO_3_^-^ were analyzed using a Thermo Scientific Dionex™ ICS-2100 Ion Chromatography System (Dionex ICS-2100). Major cations including Ca^2+^, K^+^, Mg^2+^, and Na^+^ were measured using inductively coupled plasma-optical emission spectroscopy (ICP-OES, Varian, The Netherlands). Salinity was calculated based on the NaCl concentration (weight/volume), as described before [52].

### 2.3. Microcosm Preparation

Due to dominant presence of halophilic microbes in hypersaline environments [53] and lack of information on the potential of halophiles to transform organohalogens, we strived to cultivate halophilic microbes capable of CF metabolism. Therefore, we used two media for the enrichment of halophilic bacteria and archaea: modified growth medium (MGM) and DBCM2 medium (DBC) [54]. The media were boiled and flushed with nitrogen during cooling to remove oxygen. Na_2_S·9H_2_O (0.48 g/L) was added as the reducing reagent, and resazurin (0.005 g/L) was added as redox indicator. The salinity (5%–20%) and pH (4.6–8.5) of the media were adjusted to the corresponding values measured in the sediments used as inocula (Table 1, Supplementary Table S1). Tris-base (10 mM) and acetic acid (10 mM) were used as the buffer for MGM and DBC media at high and low pH, respectively.

Initial sediment microcosms were prepared in 50 mL serum bottles containing 2.5 g wet sediment of either the top or bottom layer of the lake and 25 mL of either MGM or DBC medium. The bottles were sealed with Teflon lined butyl rubber stoppers, and the headspace was exchanged with N_2_ gas (140 kPa). CF was added to each bottle at a nominal concentration of 1.25 µmol/bottle. All cultures were set up in duplicate and incubated statically in the dark at 37 °C. Of all cultures, the sediment microcosms containing the bottom layer sediment of Lake Strawbridge in MGM with 5% salinity showed most extensive CF dechlorination and were therefore used for all subsequent experiments. Enrichment cultures were obtained by sequential transfer of the initial culture (10% (v/v)) in 120 mL bottles containing 50 mL MGM except that peptone was decreased from 5 to 0.5 g/L, yeast extract was decreased from 1 to 0.5 g/L, glycerol (10 mM) was added as a carbon source, and CF was increased to 2.5 µmol/bottle. The enrichment cultures were used to test the influence of vitamin B_12_ (0.04, 0.4, 0.8, 1.6 and 4 µM) on CF (5 µmol/bottle) transformation. Abiotic controls were performed with 4 µM vitamin B_12_, 5 µmol/bottle CF, and autoclaved (121 °C for 30 min) inoculum. In a subset of abiotic controls, Ti(III) citrate (5 mM) or dithiothreitol (DTT, 100 mM) were used as artificial electron donors [55,56]. To test CO_2_ production as a potential product of CF transformation, ^13^C-labelled CF (99%, Cambridge Isotope Laboratories, Inc., Massachusetts, USA) was added to the cultures, and ^13^CO_2_ formation was monitored as outlined below. Control cultures were prepared in parallel by supplying 100% non-labelled CF and were used for measuring natural abundance of ^13^CO_2_. The CF dechlorination rate was determined as the disappearance of CF (µmol) per day per liter of the enrichment culture (µmol/day/L) when dechlorination was stably observed. Enrichment cultures for metagenome sequencing were grown in modified MGM with and without addition of 4 µM vitamin B_12_.

### 2.4. GC Analysis

Chloromethanes were quantified from 0.2 mL headspace samples using a gas chromatograph equipped with a flame ionization detector (GC-FID, Shimadzu 2010, Kyoto, Japan) and a Stabilwax column (Cat. 10655-126, Restek Corporation, USA). The column was operated isothermally at 35 °C. Nitrogen was used as the carrier gas at a flow rate of 1 mL/min. CO, CO_2_, and methane were analyzed using a Compact GC 4.0 (Global Analyzer Solutions, Breda, The Netherlands) with a thermal conductivity detector (GC-TCD). CO and methane were measured using a Molsieve 5A column operated at 100 °C coupled to a Carboxen 1010 precolumn, and CO_2_ was measured using a Rt-Q-BOND column operated at 80 °C.

### 2.5. Isotope Analysis

^13^CO_2_ was measured in enrichment cultures containing 1.25 µmol/bottle ^13^C-labelled CF, 3.75 µmol/bottle non-labelled CF and 4 µM vitamin B_12_. The control cultures contained 5 µmol/bottle non-labelled CF and 4 µM vitamin B_12_. The carbon isotope composition of CO_2_ was determined using gas chromatography combustion isotope ratio mass spectrometry (GC/C-IRMS) consisting of a gas chromatograph (7890A Series, Agilent Technologies, Santa Clara, CA, USA) coupled via a Conflo IV interface (ThermoFinnigan, Bremen, Germany) to a MAT 253 mass spectrometer (ThermoFinnigan, Bremen, Germany). Sample separation was done with a CP-PoraBOND Q column (50 m × 0.32 mm ID, 5 µm film thickness; Agilent Technologies, Amstelveen, Netherlands) operated isothermally at 40 °C using helium as a carrier gas at a flow rate of 2 mL/min. Sample aliquots of 0.1–0.5 mL were injected at split ratios ranging from 1:10 to 1:20. The carbon isotope signatures are reported in δ notation (per mill, ‰) relative to the Vienna Pee Dee Belemnite standard.

The amount of ^13^CO_2_ produced from the ^13^C-labelled CF was expressed according to:

*δ*^13^C = (*R_sample_*/*R_standard_* − 1) × 1000 (1)

where *δ*^13^C is the ^13^C isotopic enrichment as compared to the standard (‰), *R_sample_* is the ^13^C to ^12^C ratio of CO_2_ in the sample, and *R_standard_* is the international Vienna Pee Dee Belemnite standard (VPDB, ^13^C/^12^C = 0.0112372).

### 2.6. DNA Extraction

The sediment samples collected during preparation of the sediment microcosms and kept at −80 °C were thawed and washed three times with 1.5 mL of 10 mM TE buffer (pH 7.0) to avoid interference of the high salinity with the DNA extraction as reported previously for the samples of these lakes [52]. For each sample, wet sediment (0.5 g) and the washing buffer collected using filtration through a 0.22 µm membrane filter (Millipore, MP, USA) were used for DNA extraction. DNA loss during washing was anticipated, but washing was necessary in order to extract enough DNA for further analysis [52]. DNA was extracted separately from the washed sediment and the biomass collected on the membrane filter using a PowerSoil DNA isolation kit (MO BIO, Carlsbad CA, USA) following the manufacturer’s instructions. DNA extracts from the sediment and filters were combined for each sample and used for molecular analysis. DNA of the enrichment cultures was extracted from 2 mL samples using the PowerSoil DNA isolation kit. To obtain high quality/quantity DNA for metagenome sequencing of the enrichment cultures, a MasterPure™ Gram Positive DNA Purification Kit (Epicentre, WI, USA) was used for DNA extraction from 50 mL of duplicate cultures grown with and without addition of 4 µM vitamin B_12_.

### 2.7. Quantitative PCR (qPCR)

Abundance of 16S rRNA genes of total bacteria and archaea and OHRB, including *Desulfitobacterium*, *Dehalobacter*, *Dehalococcoides*, *Sulfurospirillum*, and *Geobacter* in sediments (Lake Strawbridge) and the samples derived from the enrichment cultures were determined using qPCR. Assays were performed in triplicates using a CFX384 Real-Time system in a C1000 Thermal Cycler (Bio-Rad Laboratories, Hercules, CA, USA) with iQ^TM^ SYBR Green Supermix (Bio-Rad Laboratories, Hercules, CA, USA) as previously outlined [57]. The primers and qPCR programs used in this study are listed in Supplementary Table S2. 

### 2.8. Bacterial Community Analysis

16S rRNA gene-based bacterial community analysis was performed from sediments of Lake Strawbridge and the samples derived from the enrichment cultures. Due to the lack of CF transformation by the cultures prepared with the sediments of Lake Whurr, their bacterial community was not analyzed. The bacterial community analysis was performed as follows: a 2-step PCR was applied to generate barcoded amplicons from the V1–V2 region of the bacterial 16S rRNA genes, and the PCR products were purified and sequenced on an Illumina MiSeq platform (GATC-Biotech, currently part of Eurofins Genomics Germany GmbH, Konstanz, Germany) as described previously [58]. Primers for PCR amplification of the 16S rRNA genes are listed in Supplementary Table S2. Sequence processing was performed using NG-Tax [59]. Operational taxonomic units (OTUs) were assigned using uclust [60] in an open reference approach against the SILVA 16S rRNA gene reference database (LTPs128_SSU, version 111) [61]. Subsequently, a biological observation matrix (biom) file was generated and sequence data were further analyzed using Quantitative Insights Into Microbial Ecology (QIIME) v1.2 [62]. 

### 2.9. Metagenomic Analysis

Metagenome sequencing of duplicate enrichment cultures with and without addition of 4 µM vitamin B_12_ was performed using an Illumina HiSeq platform (PE 150 mode) at GATC Biotech. Fastp v0.19.5 [63] was used for removing adapters and low-quality reads. Assembly was done uisng metaSPAdes v3.11.1 [64] using the -meta option and the trimmed reads. This assembly was used for binning with the Metawrap v1.2 pipeline (docker version) [65]. Using the error-corrected reads from metaSPAdes, two bin sets were created from duplicate cultures with and without vitamin B_12_ with the bin_refinement module of Metawrap on binners MaxBin2 [66], MetaBat2 [67] and with Concoct [68] from the metawrap binning module [64]. The resulting two bin sets were again run through the bin_refinement module of Metawrap resulting in one bin set containing six bins and unbinned scaffolds. Raw abundance values were taken from the quant_bins module of Metawrap to calculate relative abundances per each culture. A heatmap was created with Python v3.7.3 (http://www.python.org) using pandas and seaborn. Bin quality assessment was performed with CheckM [69] for contamination and completeness, and the bins were referred to as metagenome-assembled genomes (MAGs). Taxonomic classification of the MAGs was done using pplacer [70] from CheckM and Microbial Genomes Atlas (MiGA) webserver using the TypeMat database, which contains complete/draft genomes of archaea and bacteria [71,72]. Phylogenetic analysis of the MAGs was done with MiGA and autoMLST (https://automlst.ziemertlab.com/index), and further polishing of the phylogenetic trees was performed using the Interactive Tree of Life web browser (http://itol.embl.de/) [73]. Functional annotation of the MAGs was performed using the Rapid Annotation Subsystem Technology (RAST) [74].

### 2.10. Sequence Deposition

Nucleotide sequences of bacterial 16S rRNA genes were deposited in the European Nucleotide Archive (ENA) with accession number ERS1165096–ERS1165117 under study PRJEB14107. Raw metagenome sequencing data, primary assembly, and assembled MAGs were deposited in the ENA under accession number PRJEB32090 (https://www.ebi.ac.uk/ena/data/view/PRJEB32090).

## 3. Results

### 3.1. Physical Chemical Characteristics of Sediments 

The top (0–12 cm) and bottom (>12 cm) layer sediments of Lake Strawbridge were slightly alkaline with a pH ranging from 8.2 to 8.5, whereas those of Lake Whurr were acidic with a pH of 4.6–5.4 (Table 1). Salinity, water content, and total organic carbon (TOC) were higher in the top layer compared to the bottom layer of both lake sediments (Table 1). Sodium (17.5–71.1 mg/g dry sediment) and chloride (31.9–123.5 mg/g dry sediment) were dominant among the cations and anions, respectively. Nitrate and chlorate were detected neither in the top- nor the bottom-layer sediments (Table 1).

### 3.2. CF Dechlorination in Enrichment Cultures

**Figure 1:**
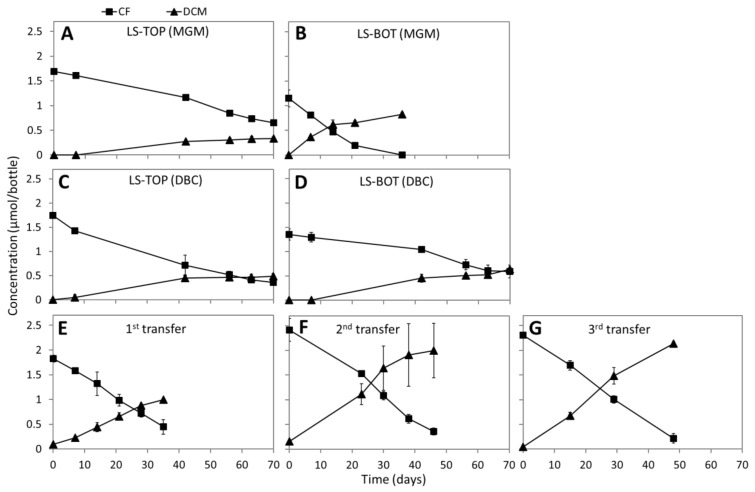
Chloroform (CF) transformation in the sediment microcosms and subsequent transfer cultures. Dechlorination of CF in modified growth medium (MGM) with top layer (LS-TOP, **A**) and bottom layer sediments (LS-BOT, **B**) from Lake Strawbridge, and dechlorination of CF in DBCM2 (DBC) medium with top (**C**) and bottom layer (**D**) sediments from the same lake. Dechlorination of CF in subsequent transfer cultures of the bottom layer sediment microcosms with MGM (**E**, **F**, **G**). Points and error bars represent the average and standard deviation of samples taken from duplicate cultures.

No CF dechlorination was observed in the sediment microcosms of Lake Whurr after 70 days of incubation, whereas CF was reductively dechlorinated to DCM in the sediment microcosms of Lake Strawbridge (Figure 1A–D). The fastest CF dechlorination rate (1.82 µmol/day/L) to DCM was observed in the microcosms with the bottom layer sediments from Lake Strawbridge in the MGM medium (Figure 1B). Therefore, this culture was selected to obtain enrichment cultures in subsequent transfers (Figure 1E–G). CM and methane as potential products of CF transformation were not detected, despite an evident lack in the mass balance between CF disappearance and DCM production in sediment microcosms (Figure 1A–D) and some enrichment transfer cultures (Figure 1E,F). The lack of methane production also suggested inhibition and/or absence of methanogens.

**Figure 2:**
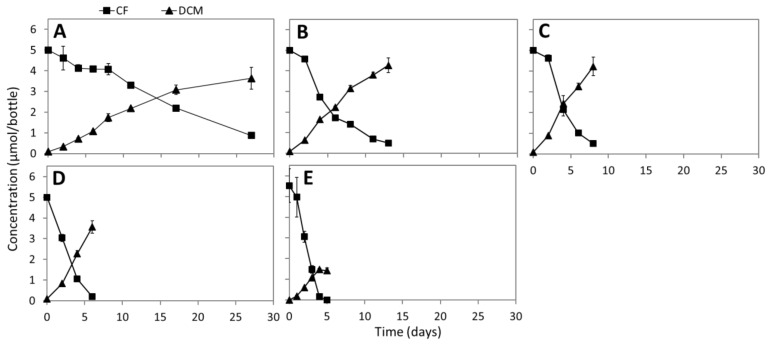
CF transformation in enrichment cultures amended with 0.04 (**A**), 0.4 (**B**), 0.8 (**C**), 1.6 (**D**), and 4 μM (**E**) vitamin B_12_. Points and error bars represent the average and standard deviation of samples taken from duplicate cultures.

Adding vitamin B_12_ at concentrations ranging from 0.04 to 4 μM steadily increased CF dechlorination rates in the enrichment cultures (Figure 2). For instance, in the cultures amended with 4 µM vitamin B_12_, the CF dechlorination rate reached 31.9 µmol/day/L (Figure 2E), ~35 times higher than the dechlorination rate in the cultures without extra vitamin B_12_ supplementation (0.9 µmol/day/L) (Figure 1E–G). In turn, increasing vitamin B_12_ concentration concurrently decreased DCM accumulation, and less than 30% of the CF was converted to DCM in the cultures amended with 4 µM vitamin B_12_ (Figure 2E). No CF dechlorination was observed in the abiotic controls even in the presence of 4 µM vitamin B_12_ (data not shown). In contrast, CF dechlorination to DCM and (or) CM was observed in abiotic controls with 4 µM vitamin B_12_ when either Ti(III) citrate or DTT were used as an artificial electron donor (Supplementary Figure S2). 

### 3.3. Analysis of ^13^CO_2_ Production from ^13^C-Labelled CF 

**Figure 3:**
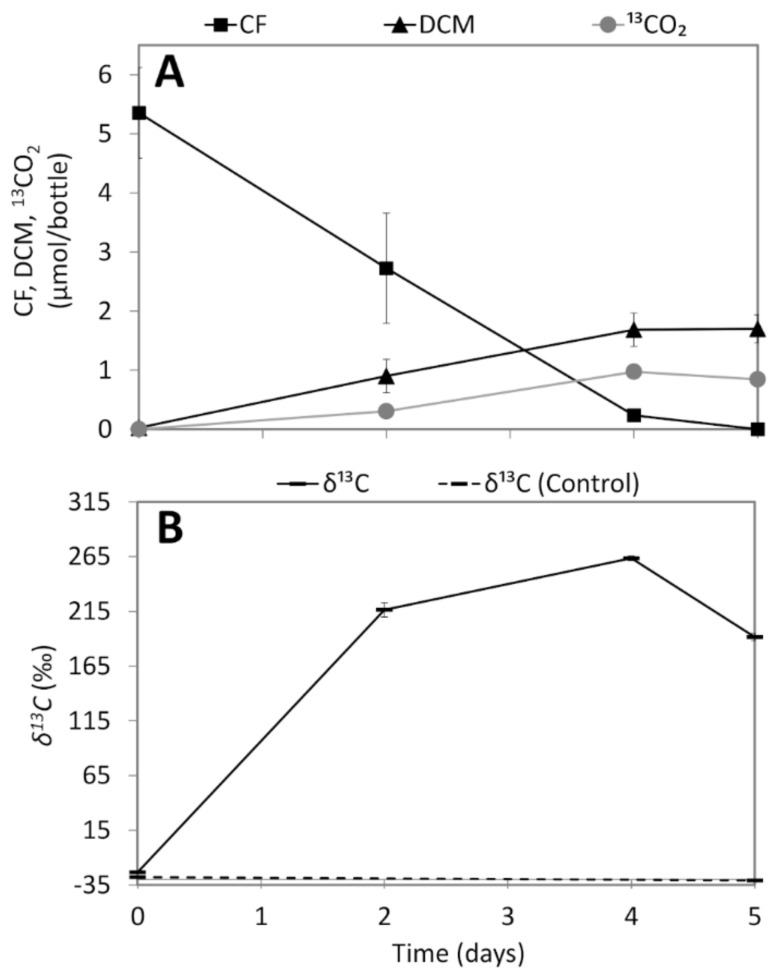
^13^CO_2_ production from CF (**A**) and δ^13^C values (**B**) in the enrichment cultures amended with 1.25 μmol/bottle ^13^C-labelled CF, 3.75 μmol/bottle non-labelled CF, and 4 μM vitamin B_12_. Control cultures contained the same concentrations of non-labelled CF and vitamin B_12_. Points and error bars represent the average and standard deviation of samples taken from duplicate cultures.

^13^CO_2_ was detected in the enrichment culture containing 1.25 µmol/bottle ^13^C-labelled CF, 3.75 µmol/bottle non-labelled CF and 4 µM vitamin B_12_ (Figure 3A). Production of ^13^CO_2_ was only detected in the culture with ^13^C-labelled CF as indicated by the increase in the *δ*^13^C value from -23.4‰ at day 0 to 263.5‰ at day 4 (Figure 3B). At day 5, 0.84 µmol/bottle ^13^CO_2_ and 1.7 µmol/bottle DCM were detected (Figure 3A). Assuming that 25% of the DCM (0.43 µmol/bottle) originated from ^13^C-labelled CF (comprising 25% of total CF mass), an ~100% ^13^C conversion of CF to CO_2_ and DCM as the main products can be inferred where removal of 1.25 µmol/bottle ^13^C-labelled CF resulted in production of 0.43 µmol/bottle ^13^C-DCM and 0.84 µmol/bottle ^13^CO_2_. 

### 3.4. qPCR and Bacterial Community Analysis

Bacterial and archaeal 16S rRNA gene copies in the top sediment layers of Lake Whurr and Lake Strawbridge were at least one order of magnitude higher than in the bottom layers of the same lakes (Supplementary Figure S3A). The top layer sediment from Lake Strawbridge had the highest number of 16S rRNA gene copies of bacteria [(3.3 ± 0.87) × 10^8^ copies/g dry sediment] and archaea [(8.6 ± 0.25) × 10^7^ copies/g dry sediment] among all the sediment samples from the two lakes (Supplementary Figure S3A). Sediment microcosms and subsequent transfer cultures prepared from the bottom layer sediment of Lake Strawbridge, contained 10^6^–10^7^ bacterial 16S rRNA gene copies/mL culture (Supplementary Figure S3B). In contrast, archaeal 16S rRNA gene copies decreased dramatically to ~10^4^ copies/mL in the sediment microcosms and to below 10^2^ copies/mL in the transfer cultures (Supplementary Figure S3B). Known OHRB including *Desulfitobacterium*, *Dehalobacter*, *Dehalococcoides*, *Geobacter*, and *Sulfurospirillum* were not detected in any of the cultures.

Bacterial community analysis based on Illumina sequencing of barcoded 16S rRNA gene fragments showed *Cyanobacteria*, *Chloroflexi*, *Proteobacteria*, and *Firmicutes* as the most predominant phyla (cumulative relative abundance > 70%) in top and bottom layer sediments of Lake Strawbridge (Supplementary Figure S4). The relative abundance of *Clostridiales* and *Halanaerobium* (*Firmicutes*) increased from 5%–16% (*Clostridiales*) and 3%–7% (*Halanaerobium*) in the bottom layer sediments to ~67% and ~18%, respectively, in the initial sediment microcosm and subsequent transfer enrichment cultures (Supplementary Figure S4).

### 3.5. Metagenomic Analysis 

**Figure 4:**
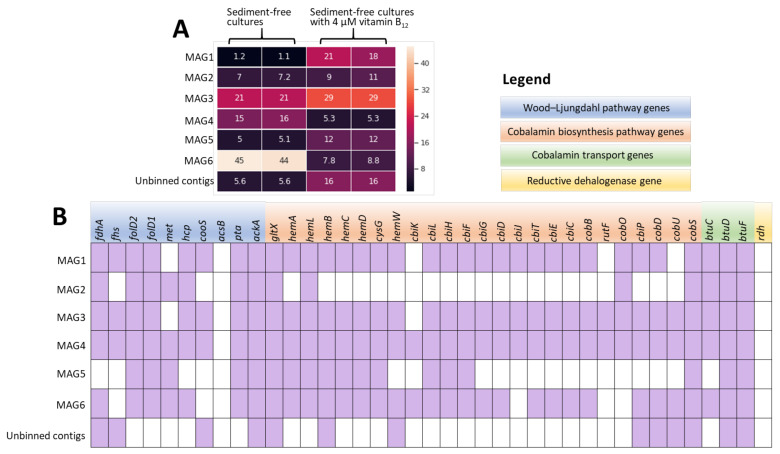
Heatmap of relative abundance (in percent) of MAGs and unbinned contigs assembled from metagenomes of the enrichment cultures with and without addition of 4 μM vitamin B_12_ (**A**) and presence and absence of genes involved in the Wood-Ljungdahl pathway, cobalamin biosynthesis, and transport and reductive dehalogenation (organohalide respiration) in the MAGs and unbinned contigs (**B**). Gene names and encoded proteins are shown in Supplementary Table S6.

A total of 50–64 million reads were obtained from sequencing of each enrichment culture with and without vitamin B_12_ (Supplementary Table S3). Six near-complete (> 95%) MAGs (MAG1–MAG6, Supplementary Table S4) were reconstructed, accounting for ~84%–95% relative abundance in the respective cultures (Figure 4A). More than 99% of the metagenomic reads of each sample were mapped to the contigs in their corresponding MAG or unbinned contigs (Supplementary Table S3). Taxonomic analysis showed that MAG2 probably belongs to a member of the genus *Halanaerobium* (*p*-value = 0.633, Supplementary Table S5), with its closest relative being *Halanaerobium saccharolyticum* DSM 6643 (average amino acid identity (AAI): 86.91%).

The remaining MAGs likely belong to novel genera based on MiGA classification (*p*-value < 0.5, Supplementary Table S5) [70,71], showing only 49%–67% AAI to their closest relatives (Supplementary Figure S5). Further, MAG6 might belong to a novel order (*p*-value = 0.452) and MAG1, MAG3, and MAG4 to novel families (*p*-value = 0.21–0.36). 

**Figure 5:**
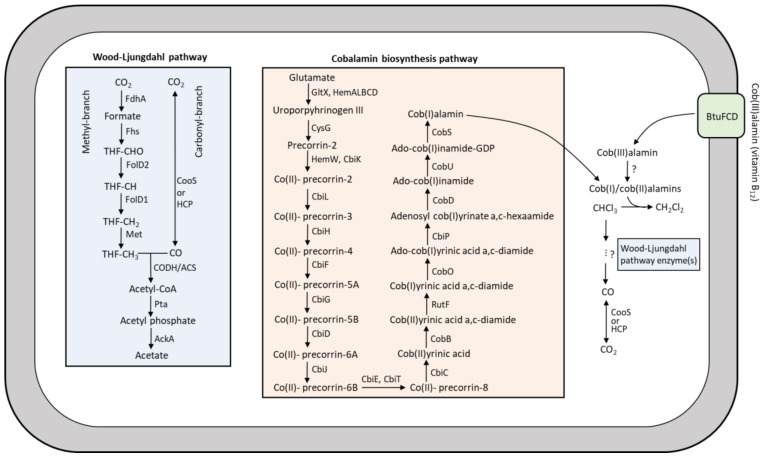
Proposed CF transformation pathway in *Clostridiales* presumably mediated by Wood-Ljungdahl pathway enzymes and cob(I)/cob(II)alamins, which are biosynthesized *de novo* or transported from the extracellular environment. The gene encoding CODH/ACS was not found in the metagenomes. Gene names and encoded proteins are shown in Supplementary Table S6.

Reductive dehalogenase genes (*rdh*) were neither detected in the MAGs nor in the unbinned contigs (Figure 4B) or the unmapped metagenomic reads. In contrast, most of the genes encoding enzymes of the WLP and cobalamin biosynthesis were identified in MAGs (Figure 4B). A notable exception was the absence of the *acs* gene cluster encoding carbon monoxide dehydrogenase/acetyl-CoA synthase (CODH/ACS) complex, the signature enzyme that connects the carbonyl- and methyl- branch of the WLP (Figure 5). Instead, a hybrid cluster protein (HCP) gene encoding a putative hydroxylamine reductase was identified in the MAGs (Figure 4B) that was recently proposed to function as CODH/ACS in the WLP [75].

## 4. Discussion 

Lake Strawbridge is a hypersaline lake with slightly alkaline pH (Table 1). A previous microbiota analysis of the lake sediment using ribosomal tag pyrosequencing of DNA and RNA revealed presence of diverse halophilic bacteria and archaea [52], and biotic chloromethane formation by the lake sediments was documented [13]. In this study, we showed CF transformation to DCM and CO_2_ by anoxic microcosms prepared from the Lake Strawbridge sediment (Figure 1,3) using a combination of anaerobic cultivation in microcosms, metabolite analyses, stable isotope labelling, molecular analyses, and genome-resolved metagenomics. Our results imply *in situ* CF removal potential in Lake Strawbridge and local halogen cycling in a hypersaline lake ecosystem. This finding is of important environmental significance considering the fact that hypersaline environments are among the hotspots of VOX formation with detrimental environmental impacts [76].

The lack of CF removal in the abiotic control cultures without artificial electron donors (Ti(III) citrate or DTT, Supplementary Figure S2) underpinned biotic CF removal in the enrichment cultures that at least needs cellular metabolism for electron donor generation. Known CF-respiring bacteria such as *Desulfitobacterium* [35,36] and *Dehalobacter* [38] were neither detected using qPCR nor 16S rRNA gene-targeted bacterial community analysis (Supplementary Figures S3 and S4). Furthermore, *rdh* were not detected in any of the MAGs, unbinned contigs (Figure 4B), or the unmapped metagenomic reads, indicating that CF respiration by OHRB was unlikely. Most OHRB harboring *rdh* genes are isolated/detected in terrestrial and aquatic environments [77], and they may lack the ability to maintain steep gradients of Na^+^ and K^+^ concentrations across their cytoplasmic membrane, which is essential for the metabolism of halophilic microorganisms in hypersaline environments [78]. Our finding indicates that even in absence of known microbes capable of metabolic CF transformation (OHRB), fortuitous biotic reactions can contribute to (partial) CF transformation and hence reduced atmospheric emission.

Members of the order *Clostridiales* were abundantly present in the sediment microcosms and enrichment cultures (Supplementary Figure S4). Acetogens belonging to this order such as members of the genera *Clostridium* and *Acetobacterium* have previously been shown to mediate co-metabolic degradation of chloromethanes [24,25]. For instance, *Acetobacterium woodii* and *Clostridium thermoaceticum* were able to degrade tetrachloromethane via CF and DCM to CO_2_ [24]. The underlying reductive pathway from tetrachloromethane to CF and DCM was proposed to be catalyzed by vitamin B_12_-dependent enzymes. This is in line with our result that CF reductive dechlorination to DCM was stimulated by adding vitamin B_12_ (< 0.8 µM, Figure 2A–C). Former research also showed that *Clostridium* species can convert vitamin B_12_ to cob(I)/cob(II)alamins, which can catalyze reductive dechlorination of CF [79,80] (Supplementary Figure S2). However, the mechanism for tetrachloromethane or CF oxidation to CO_2_, which we observed in our culture when 4 µM of vitamin B_12_ was added, is not clear. Increasing vitamin B_12_ from 0.4 to 4 µM shifted the dominant CF transformation pathway from reductive dechlorination (to DCM) to CF oxidation to CO_2_ (Figure 2,3). This finding is in line with previous studies of CF transformation using fermentative [23] and methanogenic enrichment cultures [20,22,48]. CF oxidation was proposed to occur via the net hydrolysis of CF to CO [23,24], but the enzymes involved have not been identified. Another study suggested a possible role of vitamin B_12_-dependent WLP enzyme(s) in CF hydrolysis to CO [24], which could be further oxidized to CO_2_ by CO dehydrogenase (CooS/HCP, Figure 5) [48]. Except for the *acs* gene cluster encoding CODH/ACS complex, we detected all genes encoding WLP enzymes in the *Clostridiales* MAGs. Functional WLPs were recently proposed in the absence of a full complement of genes encoding canonical WLP enzymes [75,81,82]. 

CF hydrolysis to CO was also reported by non-acetogenic and fermentative *Pantoea* spp. amended with vitamin B_12_ [23], suggesting CF hydrolysis by other (vitamin B_12_-dependent) pathways. Accordingly, we identified all genes for cobalamin biosynthesis and transport in the *Clostridiales* MAGs (Figure 4B). However, addition of external vitamin B_12_ was necessary for the enhanced reductive dechlorination and net hydrolysis of CF to DCM and CO_2_. Considering the slower CF transformation in the sediment-free enrichment cultures (Figure 1F) as opposed to the original sediment cultures (Figure 1B), these MAGs were not likely the main vitamin B_12_ producers, the abundance of which likely decreased in the sediment-free cultures during the enrichment process (Supplementary Figures S3 and S4). A possible explanation for this decline might be the previously reported CF toxicity for many vitamin B_12_-producing bacteria and archaea at concentrations as low as 0.1 µM [20], which is much lower than the CF concentrations in our enrichment cultures (2–5 µmol/bottle or 50–100 µM). Natural CF production in sediment of Lake Strawbridge was previously determined to be ~0.017 µmol/kg dry sediment [13], which may exert a negligible inhibitory effect on the vitamin B_12_/cobalamin-producing microorganisms. Cobalamin biosynthesis potential has been reported in metagenomic analyses of hypersaline aquatic and terrestrial environments [83,84]. (Enhanced) CF transformation in the presence of cobalamin indicates a key role of cobalamin not only in fulfilling important ecosystem functions such as carbon processing and gene regulation, synthesis of nucleotides and amino acids [85,86], and maintaining an abundant and diverse microbial community [83], but also potential roles in reducing CF emission to the atmosphere.

## 5. Conclusions

Hypersaline lakes are among the major sources for VOX production and emission on Earth [76]. This study showed the potential of microorganisms present in hypersaline lake sediments for co-metabolic CF transformation through vitamin B_12_-dependent pathways. Interestingly, CF transformation was only noted in microcosms prepared from the sediments of Lake Strawbridge, implying that CF natural production may have promoted VOX transformation in that lake. The MAGs obtained from CF-transforming enrichment cultures harbored the vitamin B_12_-dependant WLP pathway proposed to mediate co-metabolic CF transformation, but lacked *rdh* genes. This indicates that even in the absence of microbes/pathways capable of metabolic CF transformation, fortuitous biotic reactions can contribute to (partial) CF transformation and contribute to local halogen cycling and reducing VOX emissions to the atmosphere. 

## Figures and Tables

**Table 1:** Geochemical properties of Lake Strawbridge and Lake Whurr sediments. Duplicate sediment cores from each hypersaline lake are labelled as LS1&LS2 and LW1&LW2.

	Lake Strawbridge (LS)				Lake Whurr (LW)			
	LS1-TOP	LS2-TOP	LS1-BOT	LS2-BOT	LW1-TOP	LW2-TOP	LW1-BOT	LW2-BOT
pH ^1^	8.2	8.3	8.5	8.5	5.4	5.4	4.5	4.6
Water content (%)	37.3	27.3	16.7	15.4	26.0	25.7	24.2	23.0
Salinity (%)	17	14	5	5	15	20	11	11
TOC (g/kg dry sediment)	21	15	5	5	12	14	6	6
Na (mg/g dry sediment)	57.0	48.5	17.5	18.1	55.0	71.1	35.0	35.8
Ca (mg/g dry sediment)	0.7	0.8	0.1	0.2	6.8	4.2	0.3	0.3
K (mg/g dry sediment)	2.0	2.0	1.0	0.9	1.7	1.8	1.1	1.2
Mg (mg/g dry sediment)	2.8	2.9	1.1	1.1	4.5	4.6	3.5	3.4
Total Fe (mg/g dry sediment)	6.5	6.3	2.2	1.9	1.5	3.2	0.3	0.6
Cl^-^ (mg/g dry sediment)	101.3	84.7	31.9	33.1	93.1	123.5	64.8	64.0
SO_4_^2-^ (mg/g dry sediment)	3.9	3.6	1.5	1.8	19.6	14.8	4.3	4.4
NO_3_^-^ (mg/g dry sediment)	n.d.	n.d.	n.d.	n.d.	n.d.	n.d.	n.d.	n.d.
ClO_3_^-^ (mg/g dry sediment)	n.d.	n.d.	n.d.	n.d.	n.d.	n.d.	n.d.	n.d.

^1^ Measured in 0.01 M CaCl2 after 2 h, n.d. not detected. Abbreviations: LS, Lake Strawbridge; TOP, top layer (0–12 cm depth); BOT, bottom layer (12–24 cm depth).

## Supplementary Materials

The following are available online at https://www.mdpi.com/2076-2607/8/5/665/s1, Figure S1: Location and overview of Lake Strawbridge and Lake Whurr, Figure S2: CF transformation by vitamin B_12_ (4 µM) in MGM medium with DTT (100 mM) (A) or Ti(III) citrate (5 mM) (B) as the electron donor, Figure S3: Quantitative PCR (qPCR) targeting total bacterial and archaeal 16S rRNA genes in the top and bottom layer sediment of Lake Strawbridge and Lake Whurr (A), and sediment enrichment culture and subsequent transfer cultures derived from the bottom layer sediment microcosms of Lake Strawbridge (B), Figure S4: 16S rRNA gene based bacterial community analysis of the sediment of Lake Strawbridge and enrichment cultures, Figure S5: Phylogenetic analysis of metagenome-assembled genomes (MAGs, shown in bold), Table S1: Media components, Table S2: Primers used in this study, Table S3: Overview of metagenomic reads not mapped to the metagenome assembled-genomes (MAGs), Table S4: Features of the MAGs, Table S5: Taxonomic classification of the MAGs, Table S6. Name of the genes and encoded proteins in Figure 4 and Figure 5.

## Abbreviations

CF	chloroform
CM	chloromethane
CODH/ACS	carbon monoxide dehydrogenase/acetyl-CoA synthase
DBC	DBCM2 medium
DCM	dichloromethane
DTT	dithiothreitol
GC/C-IRMS	gas chromatography combustion isotope ratio mass spectrometry
HCP	hybrid cluster protein
MAG	metagenome-assembled genome
MGM	modified growth medium
OHRB	organohalide-respiring bacteria
RDase	reductive dehalogenase
TOC	total organic carbon
VOX	volatile organohalogens
WLP	Wood-Ljungdahl pathway
