# Supplementary material for: Metagenomic- and Cultivation-Based Exploration of Anaerobic Chloroform Biotransformation in Hypersaline Sediments as Natural Source of Chloromethanes
Source: Microorganisms. 2020 May 2;8(5):665. doi: 10.3390/microorganisms8050665 (PMC7284496; doi:10.3390/microorganisms8050665)
Supplement: Supplementary file 1 [file microorganisms-08-00665-s001.pdf]

Supplementary Materials

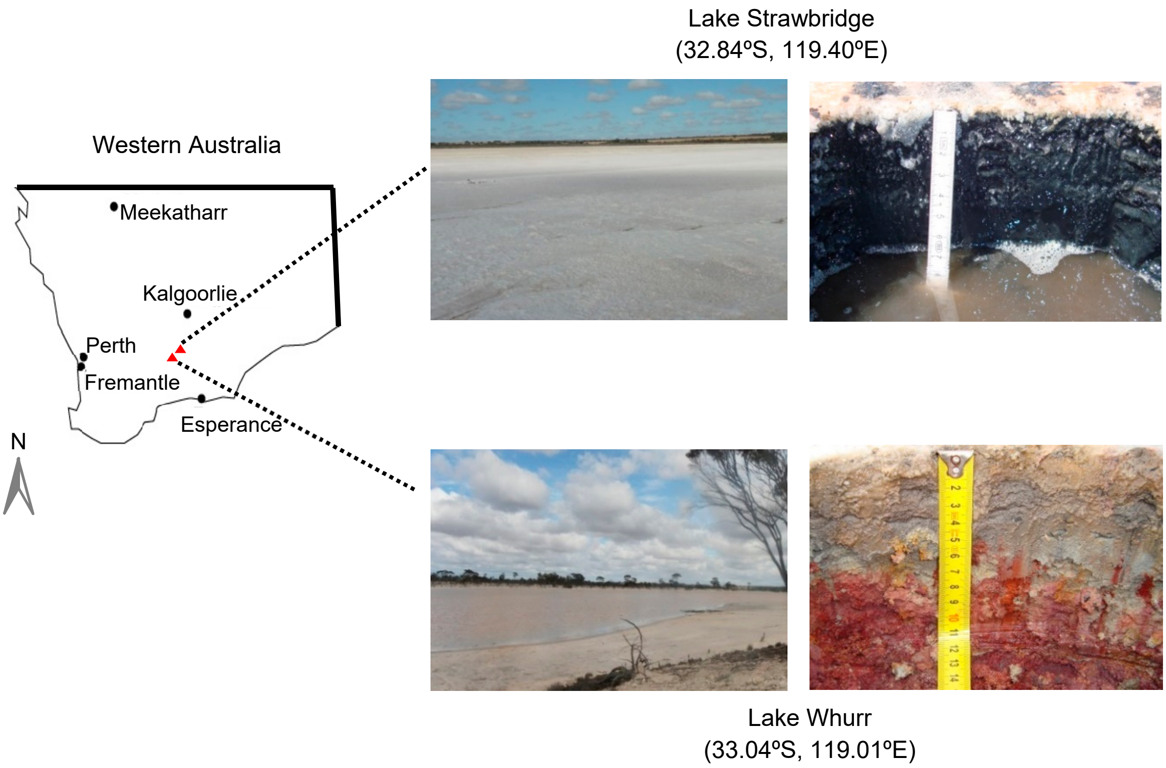

Figure S1. Location and overview of Lake Strawbridge and Lake Whurr. The coordinates of the sampling points and the depth profile are shown in the photos. The photos are a courtesy of Christoph Tubbesing from the Department of Geosciences, Universität Heidelberg.

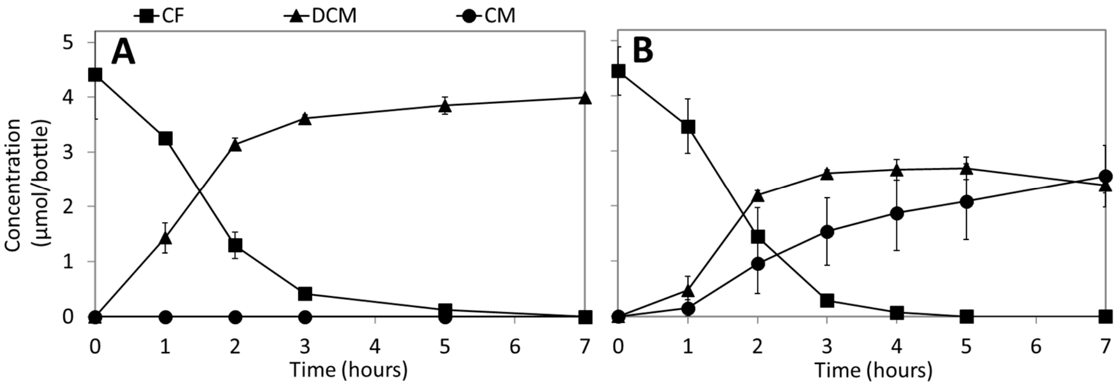

Figure S2. CF transformation by vitamin B_{12} (4 µM) in MGM medium with DTT (100 mM) (A) or Ti(III) citrate (5 mM) (B) as the electron donor. Points and error bars represent the average and standard deviation of samples taken from duplicate cultures.

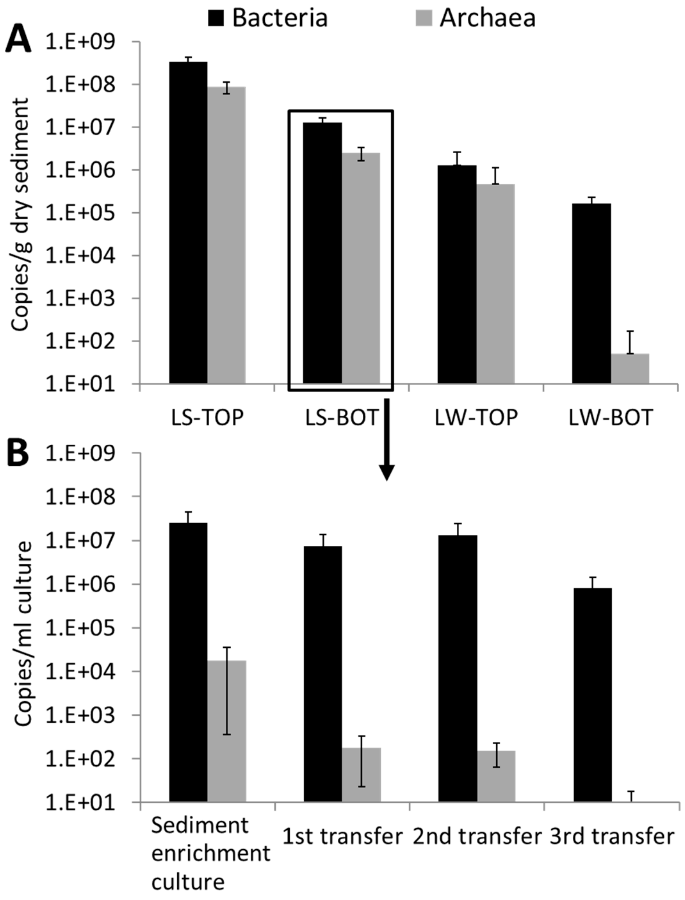

Figure S3. Quantitative PCR (qPCR) targeting total bacterial and archaeal 16S rRNA genes in the top and bottom layer sediment of Lake Strawbridge and Lake Whurr (A), and sediment enrichment culture and subsequent transfer cultures derived from the bottom layer sediment microcosms of Lake Strawbridge (B). Abbreviations: LS, Lake Strawbridge; LW, Lake Whurr; TOP, top layer (0–12 cm depth); BOT, bottom layer (12–24 cm depth). Error bars represent standard deviations of two (for enrichment samples) or four (for sediment samples) independent DNA extractions, and triplicate qPCR reactions were conducted for each DNA sample (n = 2 (4) × 3).

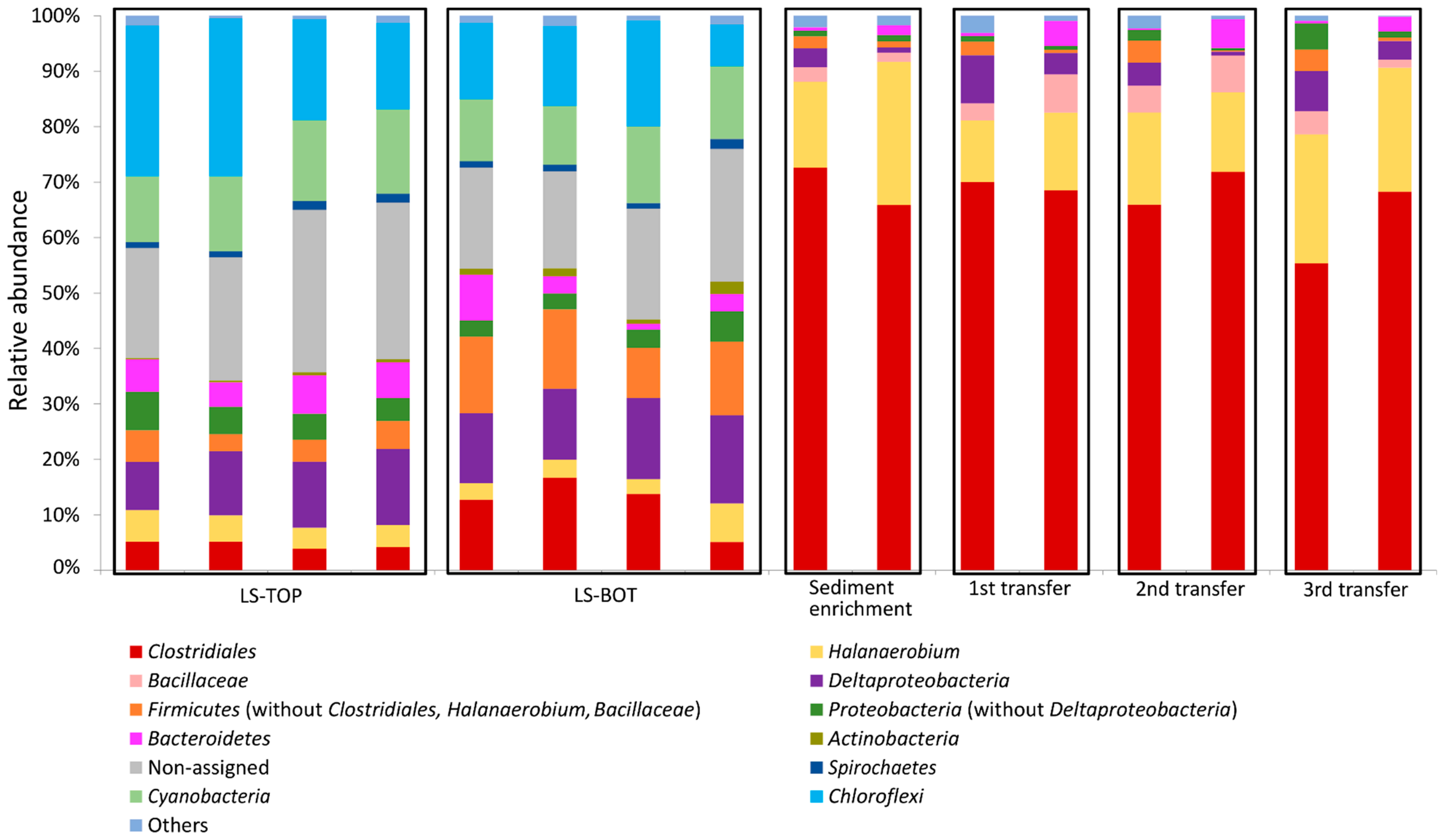

Figure S4. 16S rRNA gene based bacterial community analysis of the sediment of Lake Strawbridge and enrichment cultures. Abbreviations: LS, Lake Strawbridge; TOP, top layer (0–12 cm depth); BOT, bottom layer (12–24 cm depth). Four replicate sediment samples (LS-TOP, LS-BOT) and duplicate enrichment cultures (sediment enrichment, 1st–3rd transfers) were used for the analysis. Data are shown at phylum level, except *Deltaproteobacteria*, *Clostridiales*, *Bacillaceae* and *Halanaerobium* are shown at class, order, family and genus level, respectively. Taxa that were observed at a relative abundance below 1% were summed up and categorized as 'Others'.

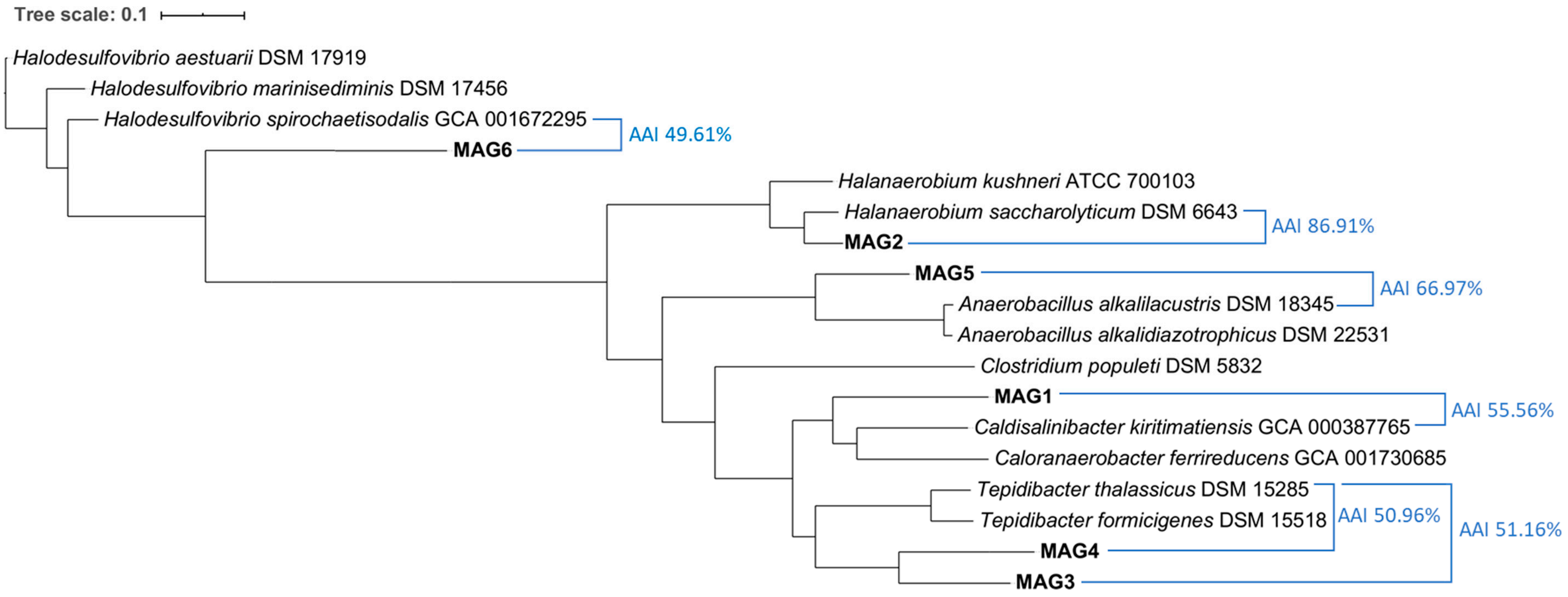

Figure S5. Phylogenetic analysis of metagenome-assembled genomes (MAGs, shown in bold). The Average Amino Acid Identity (AAI) of the MAGs and their closest relatives was indicated in the tree (shown in blue). The tree was constructed using autoMLST (https://automlst.ziemertlab.com/index). Tree scale: 10% sequence differences between genome positions.

Table S1. Media components

Medium type (Salinity %)	pH[1]	Salt water[2] (ml/L)	Demi water (ml/L)	1 M Acetic acid (ml/L)	1 M Tris-base (ml/L)	NH_4Cl (g/L)	Peptone (g/L)	Yeast extract (g/L)	Pyruvate (g/L)	Resazurin (g/L)	$Na_2S{\cdot}9H_2O$ (g/L)	SL10 trace elements[2] (ml/L)	Vit10 vitamin solution[2] (ml/L)	Cultivated sediment
MGM (20%)	5.4	660	240	10	0	0	5	1	0	0.005	0.48	0	0	LW2 TOP
MGM (17%)	8.1	560	340	0	10	0	5	1	0	0.005	0.48	0	0	LS1 TOP
MGM (15%)	5.5	500	400	10	0	0	5	1	0	0.005	0.48	0	0	LW1 TOP
MGM (14%)	8.3	460	440	0	10	0	5	1	0	0.005	0.48	0	0	LS2 TOP
MGM (11%)	4.6	360	540	10	0	0	5	1	0	0.005	0.48	0	0	LW1&2 BOT
MGM (5%)	8.5	160	740	0	10	0	5[3]	1[3]	0	0.005	0.48	0	0	LS1&2 BOT
DBC (20%)	5.4	660	240	10	0	0.5	0	0	1.1	0.005	0.48	1	3	LW2 TOP
DBC (17%)	8.1	560	340	0	10	0.5	0	0	1.1	0.005	0.48	1	3	LS1 TOP
DBC (15%)	5.5	500	400	10	0	0.5	0	0	1.1	0.005	0.48	1	3	LW1 TOP
DBC (14%)	8.3	460	440	0	10	0.5	0	0	1.1	0.005	0.48	1	3	LS2 TOP
DBC (11%)	4.6	360	540	10	0	0.5	0	0	1.1	0.005	0.48	1	3	LW1&2 BOT
DBC (5%)	8.5	160	740	0	10	0.5	0	0	1.1	0.005	0.48	1	3	LS1&2 BOT

Duplicates sediment cores from each hypersaline lake are labelled as LS-1/2TOP/BOT and LW1/2-TOP/BOT. Abbreviations: LS, Lake Strawbridge; LW, Lake Whurr; TOP, top layer (0–12 cm depth); BOT, bottom layer (12–24 cm depth).

[1]pH was adjusted to the indicated values using 10% HCl or 5 M NaOH

[2]Salt water, SL10 trace elements and Vit10 vitamin solution were prepared as described by Dyall-Smith [1]

[3]Peptone was decreased from 5 to 0.5 g/L and yeast extract was decreased from 1 to 0.5 g/L in MGM (5%) medium, and glycerol (10 mM) was added as a carbon source in the subsequent sediment-free transfer cultures

Table S2. Primers used in this study. All primers target the 16S rRNA gene, except for Unitag primers

Target	Name[1]	Oligonucleotide sequence (5′–3′)[2]	Reference for primer	Reference for PCR/qPCR program
Bacteria	27F-DegS	GTTYGATYMTGGCTCAG	[2]	
	338R–I	GCWGCCTCCCGTAGGAGT		
	338R–II	GCWGCCACCCGTAGGTGT	[3]	[4]
	Unitag1	GAGCCGTAGCCAGTCTGC		
	Unitag2	GCCGTGACCGTGACATCG	[5]	[4]
Bacteria	Eub341F	CCTACGGGAGGCAGCAG		
	Eub534R	ATTACCGCGGCTGCTGGC	[6]	[7]
Archaea	ARC787F	ATTAGATACCCSBGTAGTCC		
	ARC1059R	GCCATGCACCWCCTCT	[8]	[8]
Desulfitobacterium	Dsb406F	GTACGACGAAGGCCTTCGGGT		
	Dsb619R	CCCAGGGTTGAGCCCTAGGT	[9]	[9]
Dehalococcoides	Dco728F	AAGGCGGTTTTCTAGGTTGTCAC		
	Dco944R	CTTCATGCATGTCAAAT	[9]	[7]
Dehalobacter	Dre441F	GTTAGGGAAGAACGGCATCTGT		
	Dre645R	CCTCTCCTGTCCTCAAGCCATA	[9]	[7]
Geobacter	Geo196F	GAATATGCTCCTGATTC		
	Geo535R	TAAATCCGAACAACGCTT	[10]	[11]
Sulfurospirillum	Sulfuro114F	GCTAACCTGCCCTTTAGTGG		
	Sulfuro421R	GTTTACACACCGAAATGCGT	[12]	[12]

[1]Primer names may not correspond to original publication

[2]M = A or C; R = A or G; W = A or T; Y = C or T

Table S3. Overview of metagenomic reads not mapped to the metagenome assembled-genomes (MAGs). R1 and R1 denote replicate cultures

Sample	Total reads	Total bases	Unmapped reads	Unmapped bases	Percentage of unmapped bases (%)
Sediment-free enrichment_R1	58,152,684	8,760,115,530	309,128	11,549,894	0.13
Sediment-free enrichment_R2	51,041,562	7,685,580,260	286,440	10,741,648	0.14
Sediment-free enrichment with 4 μM B_{12}_R1	50,489,390	7,604,953,736	411,312	15,439,070	0.20
Sediment-free enrichment with 4 μM B_{12}_R2	63,749,676	9,602,843,932	562,600	21,131,032	0.22

Table S4. Features of the MAGs

MAG	Completeness (%)	Contamination (%)	GC (%)	N50 (bp)	Size (bp)
MAG1	97.9	2.9	30	50,730	4,089,467
MAG2	99.1	2.2	32	400,029	2,920,505
MAG3	100	1.8	31	115,555	3,654,683
MAG4	100	1.4	31	175,785	5,409,242
MAG5	95.6	2.0	35	94,740	3,492,562
MAG6	98.8	0.3	60	140,018	4,208,097

Table S5. Taxonomic classification of the MAGs

Bin	Domain (*p*-value[1])	Phylum (*p*-value[1])	Class (*p*-value[1])	Order (*p*-value[1])	Family (*p*-value[1])	Genus (*p*-value[1])
MAG1	*Bacteria* (0.991)	*Firmicutes* (0.961)	*Clostridia* (0.897)	*Clostridiales* (0.673)	*Clostridiaceae* (0.357)	*Caldisalinibacter* (0.162)
MAG2	*Bacteria* (0.998)	*Firmicutes* (0.991)	*Clostridia* (0.975)	*Halanaerobiales* (0.914)	*Halanaerobiaceae* (0.821)	*Halanaerobium* (0.633)
MAG3	*Bacteria* (0.983)	*Firmicutes* (0.927)	*Clostridia* (0.807)	*Clostridiales* (0.541)	*Peptostreptococcaceae* (0.214)	*Tepidibacter* (0.066)
MAG4	*Bacteria* (0.982)	*Firmicutes* (0.925)	*Clostridia* (0.801)	*Clostridiales* (0.532)	*Peptostreptococcaceae* (0.21)	*Tepidibacter* (0.064)
MAG5	*Bacteria* (0.996)	*Firmicutes* (0.982)	*Bacilli* (0.953)	*Bacillales* (0.84)	*Bacillaceae* (0.665)	*Anaerobacillus* (0.47)
MAG6	*Bacteria* (0.979)	*Proteobacteria* (0.909)	*Deltaproteobacteria* (0.761)	*Desulfovibrionales* (0.452)	*Desulfovibrionaceae* (0.188)	*Halodesulfovibrio* (0.053)

[1]The *p*-value is estimated from the empirical distribution observed in all the reference genomes of NCBI's RefSeq at each taxonomic level, and indicates the probability of the observed Average Amino Acid Identity (AAI) between genomes in the same taxon [13,14].

Table S6. Name of the genes and encoded proteins in Figures 4 and 5

Gene	Full name/encoded protein
ackA	acetate kinase gene
acsB	acetyl-CoA synthase gene
btuFCD	corrinoid transporter gene
cbiC	precorrin-8X/cobalt-precorrin-8 methylmutase gene
cbiD	cobalt-precorrin-5B (C1)-methyltransferase gene
cbiE	cobalt-precorrin-7 (C5)-methyltransferase gene
cbiF	precorrin-4/cobalt-precorrin-4 C11-methyltransferase gene
cbiG	cobalt-precorrin 5A hydrolase gene
cbiH	precorrin-3B C17-methyltransferase gene
cbiJ	precorrin-6A/cobalt-precorrin-6A reductase gene
cbiK	sirohydrochlorin cobaltochelatase gene
cbiL	precorrin-2/cobalt-factor-2 C20-methyltransferase gene
cbiP	cobyric acid synthase gene
cbiT	cobalt-precorrin-6B (C15)-methyltransferase gene
cobB	cobyrinic Acid a,c-diamide synthase gene
cobD	adenosylcobinamide-phosphate synthase gene
cobO	cob(I)alamin adenosyltransferase gene
cobS	cobalamin synthase gene
cobU	adenosylcobinamide-phosphate guanylyltransferase gene
cooS	carbon-monoxide dehydrogenase gene
cysG	uroporphyrin-III C-methyltransferase gene
fdhA	formate dehydrogenase gene
fhs	formate--tetrahydrofolate ligase gene
folD	methylenetetrahydrofolate dehydrogenase gene
gltX	glutaminyl-trna synthetase gene
hemA	glutamyl-trna reductase gene
hemB	porphobilinogen synthase gene
hemC	hydroxymethylbilane synthase gene
hemD	uroporphyrinogen-III synthase gene
hemL	glutamate-1-semialdehyde 2,1-aminomutase gene
hemW	heme chaperone gene
met	methylenetetrahydrofolate reductase gene
pta	phosphate acetyltransferase gene
rdh	reductive dehalogenase gene
rutF	FMN reductase gene

References

1. Dyall-Smith, M. *The Halohandbook: protocols for haloarchaeal genetics*; Haloarchaeal Genetics Laboratory, Melbourne, 2008; Vol. 14.
2. van den Bogert, B.; de Vos, W.M.; Zoetendal, E.G.; Kleerebezem, M. Microarray analysis and barcoded pyrosequencing provide consistent microbial profiles depending on the source of human intestinal samples. *Appl. Environ. Microbiol.* **2011**, *77*, 2071-2080.
3. Daims, H.; Brühl, A.; Amann, R.; Schleifer, K.-H.; Wagner, M. The domain-specific probe EUB338 is insufficient for the detection of all Bacteria: development and evaluation of a more comprehensive probe set. *Syst. Appl. Microbiol.* **1999**, *22*, 434-444.
4. Lu, Y.; Ramiro-Garcia, J.; Vandermeeren, P.; Herrmann, S.; Cichocka, D.; Springael, D.; Atashgahi, S.; Smidt, H. Dechlorination of three tetrachlorobenzene isomers by contaminated harbor sludge-derived enrichment cultures follows thermodynamically favorable reactions. *Appl. Microbiol. Biotechnol.* **2017**, *101*, 2589-2601.
5. Tian, L.; Scholte, J.; Borewicz, K.; Bogert, B.; Smidt, H.; Scheurink, A.J.; Gruppen, H.; Schols, H.A. Effects of pectin supplementation on the fermentation patterns of different structural carbohydrates in rats. *Mol. Nutr. Food Res.* **2016**, *60*, 2256-2266.
6. Muyzer, G.; De Waal, E.C.; Uitterlinden, A.G. Profiling of complex microbial populations by denaturing gradient gel electrophoresis analysis of polymerase chain reaction-amplified genes coding for 16S rRNA. *Appl. Environ. Microbiol.* **1993**, *59*, 695-700.
7. Atashgahi, S.; Maphosa, F.; Doğan, E.; Smidt, H.; Springael, D.; Dejonghe, W. Small-scale oxygen distribution determines the vinyl chloride biodegradation pathway in surficial sediments of riverbed hyporheic zones. *FEMS Microbiol. Ecol.* **2013**, *84*, 133-142.
8. Yu, Y.; Lee, C.; Kim, J.; Hwang, S. Group-specific primer and probe sets to detect methanogenic communities using quantitative real-time polymerase chain reaction. *Biotechnol. Bioeng.* **2005**, *89*, 670-679.
9. Smits, T.H.; Devenoges, C.; Szynalski, K.; Maillard, J.; Holliger, C. Development of a real-time PCR method for quantification of the three genera *Dehalobacter, Dehalococcoides*, and *Desulfitobacterium* in microbial communities. *J. Microbiol. Methods* **2004**, *57*, 369-378.
10. Amos, B.K.; Sung, Y.; Fletcher, K.E.; Gentry, T.J.; Wu, W.-M.; Criddle, C.S.; Zhou, J.; Löffler, F.E. Detection and quantification of *Geobacter lovleyi* strain SZ: implications for bioremediation at tetrachloroethene-and uranium-impacted sites. *Appl. Environ. Microbiol.* **2007**, *73*, 6898-6904.
11. Azizian, M.F.; Marshall, I.P.; Behrens, S.; Spormann, A.M.; Semprini, L. Comparison of lactate, formate, and propionate as hydrogen donors for the reductive dehalogenation of trichloroethene in a continuous-flow column. *J. Contam. Hydrol* **2010**, *113*, 77-92.
12. Sutton, N.B.; Atashgahi, S.; Saccenti, E.; Grotenhuis, T.; Smidt, H.; Rijnaarts, H.H. Microbial community response of an organohalide respiring enrichment culture to permanganate oxidation. *PLoS One* **2015**, *10*, e0134615.
13. Rodriguez-R, L.M.; Gunturu, S.; Harvey, W.T.; Rosselló-Mora, R.; Tiedje, J.M.; Cole, J.R.; Konstantinidis, K.T. The Microbial Genomes Atlas (MiGA) webserver: taxonomic and gene diversity analysis of Archaea and Bacteria at the whole genome level. *Nucleic Acids Res.* **2018**, *46*, W282-W288.
14. Rodriguez-R, L.M.; Gunturu, S.; Tiedje, J.M.; Cole, J.R.; Konstantinidis, K.T. Nonpareil 3: fast estimation of metagenomic coverage and sequence diversity. *mSystems* **2018**, *3*, e00039-00018.
